# Chest compressions at altitude are of decreased quality, require more effort and cannot reliably be self-evaluated

**DOI:** 10.1186/s13049-023-01149-y

**Published:** 2023-12-04

**Authors:** Michiel J. van Veelen, Hermann Brugger, Marika Falla, Giacomo Strapazzon

**Affiliations:** 1 Institute of Mountain Emergency Medicine, Eurac Research, Via Ipazia 2, Bolzano, 39100 Italy; 2 Department of Sport Science, Medical Section, University of Innsbruck, Innsbruck, Austria; 3 International Commission for Mountain Emergency Medicine (ICAR MEDCOM), Kloten, Switzerland; 4 Department of Neurology/Stroke Unit, Hospital of Bolzano (SABES-ASDAA), Bolzano, Italy

To the Editor,

We read with interest the recent study by Niederer et al. about the influence of altitude (3454 m) on physical exhaustion during cardiopulmonary resuscitation (CPR) [1]. The authors analyze a secondary objective of a previous study that showed a reduction of chest compression (CC) depth at altitude [2] and show that heart rates are significantly higher at altitude before and after CPR. Such results support the results of a study that we recently published [3] as well one of Sato et al. [4], where we both observed a significant decrease in peripheral oxygen saturation, an increase in heart rate, as well as an increase in fatigue during CPR at 3000 and 5000 m, and 3700 m, respectively. Conversely to Niederer et al., the forty-eight participants in our study, all helicopter emergency medical services (HEMS) providers, reached altitudes without physical effort simulating a rapid helicopter ascent in a hypobaric chamber [3]. Nevertheless, we also observed an increase in heart rate during CPR that was even bigger compared to the one observed by Niederer et al. and was dependent on the altitude reached.

We did not investigate exhaustion but instead effort during CC. In our study the subjective effort, reported on a visual analogue scale (VAS), was significantly higher at 5000 m than at 200 and 3000 m [3]. As suspected by Niederer et al., we also showed that providers were not able to reliably self-evaluate the quality of CC at altitude.

Overall, the results of Niederer et al. [1, 2], as well as ours [3], show a greater effort and an impairment of the ability of providers to comply to resuscitation guidelines at altitude. Such impairment starts after 60 to 90 s of CC even in resting providers, so there is a significant risk that the depth of CCs can drop below the recommended 50 mm already before 2 min, when switching the CC provider is recommended by international CPR guidelines [5]. We suggest that apart from ventilatory pauses, more frequent rotations and routine use of mechanical chest compression devices could be of help in overcoming exhaustion and CPR quality decrease both in ground and air missions. We suggest reevaluation of CPR guidelines for providers practicing at altitudes of 3000 m and higher [5].
